# The role of a structured exercise training program on cardiac structure and function after acute myocardial infarction: study protocol for a randomized controlled trial

**DOI:** 10.1186/s13063-015-0612-6

**Published:** 2015-03-12

**Authors:** Ricardo Fontes-Carvalho, Francisco Sampaio, Madalena Teixeira, Vasco Gama, Adelino F Leite-Moreira

**Affiliations:** Cardiology Department, Gaia Hospital Centre, Rua Conceicao Fernandes, 4434-502 Vila Nova Gaia, Portugal; Department of Physiology and Cardiothoracic Surgery, Faculty of Medicine, University of Porto, Al. Prof. Hernâni Monteiro, 4200 - 319 Porto, Portugal; Department of Cardiothoracic Surgery, Centro Hospitalar São João, Al. Prof. Hernâni Monteiro, 4200 - 319 Porto, Portugal

**Keywords:** Diastole, Systole, Exercise therapy, Myocardial infarction

## Abstract

**Background:**

Exercise training is effective in improving functional capacity and quality of life in patients with coronary artery disease, but its effects on left ventricular systolic and diastolic function are controversial. Diastolic dysfunction is a major determinant of adverse outcome after myocardial infarction and, contrary to systolic function, no therapy or intervention has proved to significantly improve diastolic function. Data from animal studies and from patients with diastolic heart failure has suggested that exercise training can have a positive effect on diastolic function parameters.

This trial aims to evaluate if a structured exercise training program can improve resting left ventricular diastolic and systolic function in patients who have had an acute myocardial infarction.

**Methods/Design:**

This is a phase II, prospective, randomized, open-label, blinded-endpoint trial that will include at least 96 consecutive patients who have had an acute myocardial infarction one month previously. Patients will be randomized (1:1) to an exercise training program or a control group, receiving standard of care. At enrolment, and at the end of the follow-up period, patients will be submitted to an echocardiography (with detailed assessment of diastolic and systolic function using recent consensus guidelines), cardiopulmonary exercise testing, an anthropometric assessment, blood testing, and clinical evaluation. Patients randomized to the intervention group will be submitted to an eight-week outpatient exercise program, combining endurance and resistance training, for three sessions per week. The primary endpoint will be the change in lateral E’ velocity immediately after the eight-week exercise training program. Secondary endpoints will include other echocardiographic parameters of left ventricular diastolic and systolic function, cardiac structure, metabolic and inflammation biomarkers (high-sensitivity C-reactive protein and pro-BNP), functional capacity (peak oxygen consumption and anaerobic threshold) and anthropometric measurements.

**Discussion:**

New strategies that can improve left ventricular diastolic function are clinically needed. This will be the first trial to evaluate, in patients who have had an acute myocardial infarction, the effects of a structured program of exercise training on diastolic and systolic function, assessed by novel echocardiographic parameters.

**Trial registration:**

Registered with ClinicalTrials.gov (reference: NCT02224495) on 21 August 2014.

## Background

Exercise training is effective in improving functional capacity and quality of life in patients with coronary artery disease [1,2]. It is known that exercise can improve several cardiovascular and non-cardiovascular parameters, such as glucose metabolism, skeletal muscle function, oxidative stress, vascular function, pulmonary circulation, ischemia-reperfusion lesion, and ventricular remodelling [3].

However, the effect of exercise training on left ventricle systolic and diastolic function is still controversial [4-6], especially after acute myocardial infarction (AMI), where no longitudinal study has evaluated cardiac function using modern echocardiographic parameters, such as those derived from tissue Doppler analysis. After AMI, the effect of exercise training on diastolic function can be clinically relevant because the majority of these patients have diastolic dysfunction (DD) [4,7,8] and, most importantly, because no therapy or intervention has been shown to significantly improve diastolic function [9]. Data from animal studies are encouraging, showing that endurance training can improve myocardial relaxation and calcium homeostasis by increasing the myocardial expression of *SERCA2a* and phospholamban (*PLB*) [10,11]. Also, a recent study evaluating patients with heart failure with preserved ejection fraction (also known as diastolic heart failure) has shown that the combination of endurance and resistance training can improve diastolic function parameters [12]. Finally, it is also still controversial whether systolic function can be improved by exercise training [4,13,14]. Most of these studies were small, lacked control groups, and evaluated systolic function only by ejection fraction, which has several limitations in the evaluation of global left ventricle systolic function [15].

In this trial our aim is to assess, in patients who have had an AMI, if a structured exercise training program can improve resting left ventricular systolic and diastolic function.

## Methods/Design

### Trial design

**Figure 1:**
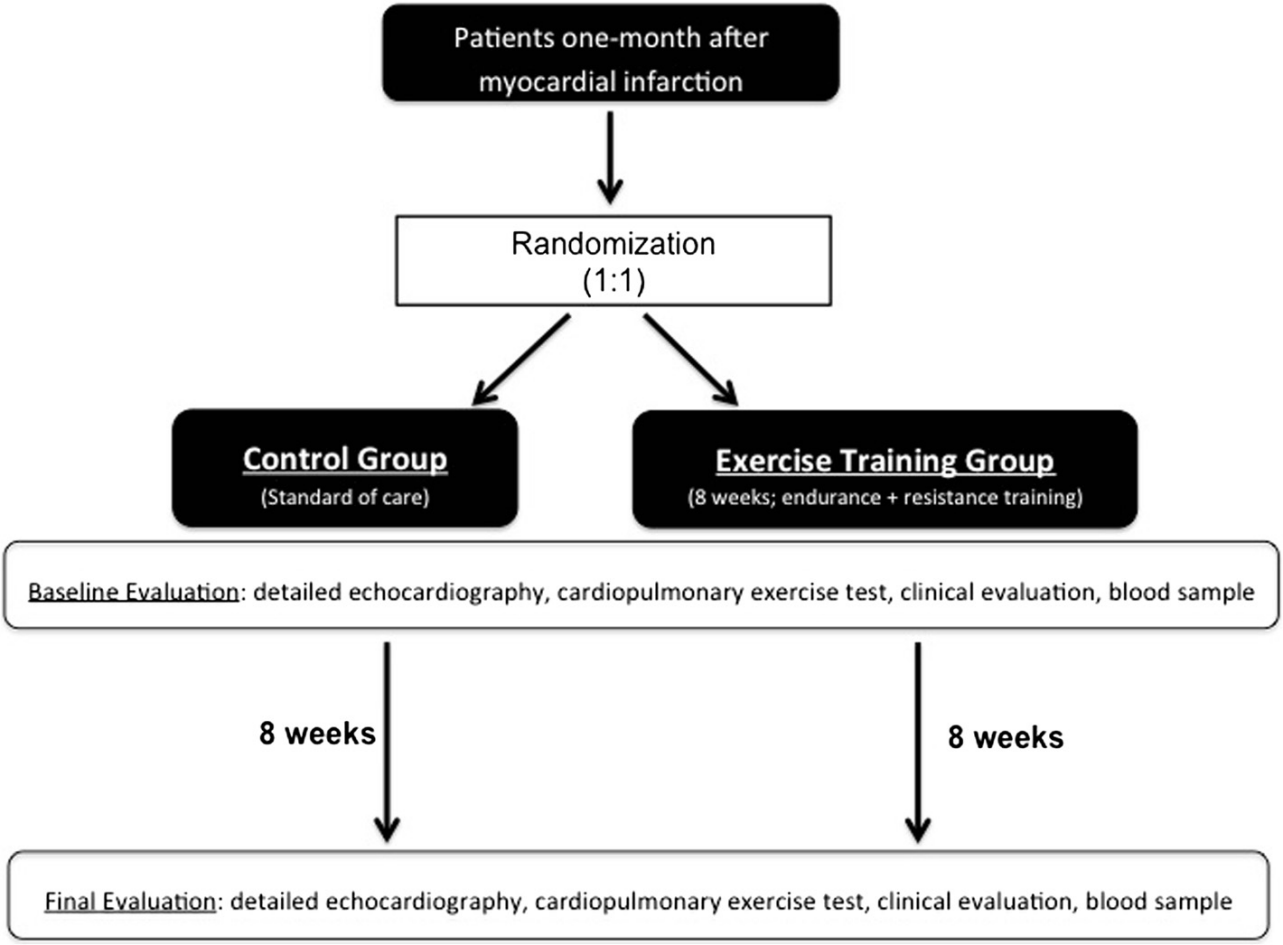
**Design of the trial.** In this prospective, randomized, open-label, blinded-endpoint trial consecutive patients who have had an acute myocardial infarction one month previously will be randomized (1:1) to an eight-week outpatient exercise training program or standard of care. The primary endpoint will be the change in lateral E’ velocity between baseline and follow-up.

In this prospective, randomized, open-label, blinded-endpoint (**“The Effect of Exercise Training on Cardiac Structure and Function”**) trial, patients who have had an AMI one month previously will be randomized (1:1) to be included in an eight-week duration exercise training program or a control group, receiving standard of care. Immediately before enrolment and at the end of the follow-up period, patients will be submitted to a clinical evaluation, detailed echocardiography, cardiopulmonary exercise testing, and an anthropometric assessment, according to the study design outlined in Figure 1. Because the intervention is a structured exercise training program, it is not possible to perform patient blinding. However, the medical staff performing all the measurements will be blinded to the type of intervention.

Randomization by blocks will be used with an allocation sequence based on a fixed block size of eight, generated with a computer random number generator.

### Participants

Eligible patients will be consecutive individuals admitted to Gaia Hospital Centre (a tertiary care hospital with a reference population of 700,000 patients) after AMI, including both ST-elevation and non-ST elevation myocardial infarction. For inclusion in the study, we will use the criteria in the universal definition of myocardial infarction [16]. Table 1 details the study’s inclusion and exclusion criteria.

**Table 1:** **Study population: inclusion and exclusion criteria**

**Inclusion criteria:**	
Between 18 and 75-years-old	
Acute myocardial infarction (according to the universal definition of myocardial infarction [16])	
Both ST elevation and non-ST elevation myocardial infarction	
One month since discharge	
Left ventricular ejection fraction >30%	
Able to exercise	
**Exclusion criteria**	
Moderate or severe valvular disease	
Atrial fibrillation	
Uncontrolled atrial or ventricular tachyarrhythmias	
Exercise-induced myocardial ischemia	
Pericardial disease	
Moderate or severe chronic lung disease (vital capacity and/or forced expiratory volume in 1 second <80% of age-dependent predicted value)	
Severe renal disease or dysfunction (creatinine clearance <30 mL/min, calculated by the Cockcroft-Gault formula)	
Anaemia (hemoglobin <12 g/dL)	

### Intervention: the exercise training program

Patients randomized to the exercise training group will be included in an eight-week outpatient program, encompassing three sessions per week, combining endurance and resistance training. Each session will consist of 10 minutes of warm up, 50 minutes of aerobic and resistance training, and 10 minutes of cool down. During the eight weeks, endurance training (cycling in the first four weeks and treadmill in the remaining four weeks) of increasing intensity will be performed. Training intensity will be individualized to a target heart rate of 70 to 85% of the maximal heart rate achieved in the baseline cardiopulmonary exercise test. Resistance training will also be included in each session consisting of arm, leg, and thoracic exercises, including dumbbell or weight training depending, on the patient’s condition and exercise capacity. Clinical and training parameters will be monitored throughout the exercise including arterial blood pressure, heart rate, glycaemia (in diabetic patients), and training level intensity. Fatigue will be monitored using the Borg scale.

Patients randomized to the control group will receive standard of care, with regular appointments with the cardiologist, optimized medication, and recommendations on healthy lifestyle.

### Measurements: the echocardiographic evaluation protocol

A single experienced cardiologist, blinded to the patient assignment group, will perform all echocardiographic studies using an ultrasound system (iE33, Philips Medical Solutions, Best, The Netherlands) equipped with an S5-1 and X5-1 transducer. Cardiac chamber dimensions, volumes, and left ventricular mass will be measured according to current recommendations and indexed to body surface area [17]. Mitral inflow velocities will be accessed using pulsed-wave Doppler in the apical four-chamber view, placed between the tips of the mitral leaflets and velocities recorded at end-expiration. Tissue Doppler velocities will also be acquired at end-expiration, in the apical four-chamber view, with the sample positioned at the septal and lateral mitral annulus for determination of systolic (S’), early diastolic (E’), and late diastolic (A’) velocities. Pulsed wave Doppler velocities at the upper right pulmonary vein will also be recorded to calculate the Ard-Ad relation: the time difference between the duration of the atrial reverse wave of the pulmonary flow (Ard) and the mitral A-wave duration. For all parameters, the average of three consecutive heart beats will be recorded.

Left ventricle diastolic function will be evaluated according to the EAE/ASE consensus guidelines on diastolic function evaluation [18], which include the determination of peak early (E) and late (A) diastolic mitral inflow velocities, deceleration time of early left ventricular filling (DT), E/A ratio, isovolumetric relaxation time (IVRT), myocardial early diastolic velocities at the septal and lateral side of mitral annulus (E’ septal, E’ lateral, and E’ mean), E/E’ ratio (including septal, lateral, and mean E/E’) and Ard-Ad relation. Moreover, using the consensus criteria [18], patients will be categorized in DD grades: normal diastolic function, grade I (mild DD), grade II (moderate DD), and grade III (severe DD), by two blinded independent cardiologists. In case of discordance, each case will be discussed individually.

Left ventricle systolic function will be evaluated by calculation of biplane ejection fraction (Simpson’s rule) and determination of systolic velocities at the septal and lateral side of mitral annulus by tissue Doppler (S’ septal and S’ lateral). It is known that, compared to ejection fraction, S’ velocities are more sensitive parameters for global and regional systolic function evaluation after myocardial infarction [19].

### Measurements: cardiopulmonary exercise testing

At the beginning and end of the study protocol, patients will be submitted to symptom-limited cardiopulmonary exercise testing on a treadmill, using the modified Bruce protocol (Cardiovit CS-200 Ergo Spiro; Schiller, Baar, Switzerland). Expired gases will be continuously collected throughout exercise and analyzed for ventilatory volume (VE) and for oxygen (O_2_) and carbon dioxide (CO_2_) content, using dedicated analyzers. Standard spirometry (forced expiratory volume in one second (FEV1) and forced vital capacity (FVC)) will also be performed before the exercise test. Equipment calibration and all measurements will be done according to the recommendations of the American Thoracic Society and American College of Chest Physicians [20]. The following variables will be calculated: peak oxygen consumption (pVO_2_), measured in milliliter per kilogram per minute (mL/Kg/min); peak respiratory exchange ratio, defined by the ratio of CO_2_ production to O_2_ consumption at peak effort; oxygen consumption at anaerobic threshold (AT), defined as the point at which CO_2_ production increases disproportionately in relation to O_2_ consumption, obtained from a graph plotting O_2_ consumption against CO_2_ production; and total exercise duration (seconds). It is known that cardiopulmonary exercise testing with O_2_ consumption measurement provides the most accurate, reliable, and reproducible measurements of exercise capacity [21]. We will assess exercise capacity mainly by measuring pVO_2_, which is considered the gold standard parameter for functional capacity assessment [22]. However, we will also evaluate submaximal exercise capacity by determination of the ventilatory AT that has the advantage of being relatively effort independent [21].

### Measurements: clinical, analytical, and anthropometric data

At baseline and at the end of the study protocol all patients will be submitted to a clinical evaluation performed by a cardiologist, a blood sample, and detailed anthropometric evaluation. The clinical evaluation will include an interview, review of medical registries, and a physical examination to collect data on cardiovascular risk factors, previous medical history, medication use or change since last evaluation, symptoms of angina, and New York Heart Association functional status (NYHA class).

The anthropometric evaluation will include the measurement of height, weight, waist circumference, and hip circumference. Body mass index ((BMI) weight/height^2^ in kg/m^2^) will be calculated for each subject. Waist circumference will be measured at the midpoint between the iliac crest and the lower rib margins, measured in the midaxillary line. Body composition will be assessed by bioelectrical impedance analysis (Tanita Inner Scan BC-522; Tanita, Tokyo, Japan) to determine body fat percentage (%).

A fasting venous blood sample will be obtained immediately before and after the study for measurement of glucose, total cholesterol, LDL-cholesterol, HDL-cholesterol, triglycerides, high-sensitivity C-reactive protein, and N-terminal pro-BNP. Insulin resistance will be assessed using the Homeostasis Model Assessment of Insulin Resistance (HOMA-IR) score.

### Study endpoints

The primary endpoint will be the change in E’ lateral velocity after the eight-week exercise training program. Secondary endpoints will be other echocardiographic parameters of left ventricular diastolic and systolic function, metabolic and inflammation biomarkers, functional capacity, and anthropometric measurements, as detailed in Study primary and secondary endpoints:

- **Primary endpoint**
- E’ lateral velocity: change between baseline and follow-up
- **Secondary endpoints**
– Left ventricular diastolic function parameters:
- E’ septal velocity; E/E’ septal, lateral and mean ratio; E/A ratio; E-wave deceleration time; isovolumetric relaxation time; diastolic dysfunction grades according to the ASE/ESE consensus
– Systolic function parameters:
- Ejection fraction, S’ lateral and septal velocities
– Functional capacity parameters:
- Peak VO_2_; VO_2_ at anaerobic threshold; exercise duration
– Metabolic biomarkers:
- Insulin and glucose plasma levels; insulin resistance (Homeostasis Model Assessment);
– Cardiovascular biomarkers:
- N-terminal pro-BNP and high sensitivity C-reactive protein
– Anthropometric parameters:
- Weight, body mass index, waist and hip perimeter, fat mass percentage by bioimpedance analysis

### Statistical analysis and sample size calculation

The analysis of the primary endpoint will be performed on an intention-to-treat basis by repeated-measures analysis of covariance (ANCOVA), including the following variables: baseline E′ velocity, age, mean arterial pressure, treatment group, gender, and baseline degree of DD.

To calculate the sample size we used as a reference the data from the study of Edelman *et al*., which tested the effect of exercise training in patients with heart failure with preserved ejection fraction [12]. Assuming a standard deviation of the change in E’ velocity from baseline to follow-up of 1.5 cm/s in each group, we estimated that, to detect a difference of 1 cm/s in the change of E’ velocity between treatment groups, a minimum of 48 patients would be required in each group. A significance level of 5% and a statistical power of 90% will be defined.

### Ethical and legal issues

Informed consent will be obtained from all patients prior to study beginning. The ethics review board (*Comissão de Ética do Centro Hospitalar de Vila Nova de Gaia*) approved the study protocol (reference: 627/10). The study conforms to the principles outlined in the Declaration of Helsinki (1975) and has been registered at ClinicalTrials.gov (reference: NCT02224495).

## Discussion

Until now no therapy or intervention has proved to significantly improve diastolic function and to change the prognosis of diastolic heart failure [9,23]. Structured programs of exercise training can have several benefits in metabolic, muscular, pulmonary, and cardiovascular parameters. Data from experimental studies also suggest that exercise training can improve systolic and diastolic function and promote favorable myocardial remodelling [10,11]. In this study, we aim to evaluate the impact of a structured program of endurance plus resistance training on cardiac function in patients who have had an AMI. Theoretically, the benefits of exercise training can depend on clinical context and on the type, intensity, and duration of exercise [24,25]. In this study, we will test a combination of endurance plus resistance training during eight-weeks, encompassing three sessions per week. The combination of endurance and strength training is important because strength training is useful to accelerate the improvements in skeletal muscle bulk and function. The protocol that will be used is similar to the one applied by Edelmann *et al*., that also tested the effect of exercise training on diastolic function in patients with heart failure with preserved ejection fraction [12].

Cardiac function will be assessed by echocardiography using both traditional parameters and those derived from tissue Doppler imaging. Diastolic function will be evaluated according to the latest consensus criteria between the American Society of Echocardiography and the European Association of Echocardiography. For the evaluation of left ventricular diastolic function it is known that traditional parameters, based on mitral inflow and pulmonary vein flow velocities (such as the E/A ratio, DT, or Ard-Ad), are markedly influenced by loading conditions, left ventricular compliance, and left atrium function [18,23,26,27]. Therefore the primary endpoint in this study will be the E’ velocity, determined by tissue Doppler analysis. Several studies have shown that E’ velocity is closely related with left ventricular relaxation assessed invasively by tau (the time constant of isovolumic pressure decline) [28,29]. We will also evaluate the E/E’ ratio, which is a diastolic function parameter derived from tissue Doppler that closely reflects left ventricular filling pressures determined invasively [18,30]. Systolic function will be evaluated by ejection fraction and by systolic mitral annulus velocities (S’ velocity) from tissue Doppler. It is known that ejection fraction is both preload- and afterload-dependent, and has several limitations in the assessment of global left ventricle systolic function. On the contrary, the evaluation of S’ velocities is more sensitive in the evaluation of global and regional systolic function after myocardial infarction [19].

## Trial status

Recruitment for the study has finished; 188 patients have been included as of February 2015.

## Abbreviations

AMI: Acute myocardial infarction
ASE: American Society of Echocardiography
AT: Anaerobic threshold
BMI: Body mass index
BNP: Brain Natriuretic Peptide
DD: Diastolic dysfunction
DT: Deceleration time
EAE: European Association of Echocardiography
IVRT: Isovolumetric relaxation time
HOMA-IR: Homeostasis Model Assessment of Insulin Resistance
pVO_2_: Peak oxygen consumption
